# The impact of combining regional nerve block with general anesthesia on cognitive function in patients undergoing elbow joint release surgery: a randomized controlled trial

**DOI:** 10.1097/JS9.0000000000002717

**Published:** 2025-06-12

**Authors:** Fang Xie, Ai-Hua Qi, Fan Pan, Ying Zhang, Ning Gan, Xiao-Tao Xu, Ai-Zhong Wang, Nan-Nan Zhang

**Affiliations:** Department of Anesthesiology, Shanghai Sixth People’s Hospital Affiliated to Shanghai Jiao Tong University School of Medicine, Shanghai, China

**Keywords:** cognitive function, elbow joint surgery, regional nerve block

## Abstract

**Background and aim:**

Regional nerve block, as an anesthetic technique, can enhance postoperative recovery for patients. Postoperative cognitive dysfunction (POCD) remains a critical concern for patients undergoing elbow joint release surgery. This randomized controlled trial evaluated whether combining regional nerve block (RNB) with general anesthesia (GA) improves cognitive outcomes compared to GA alone.

**Methods:**

A single-center and single-blind (outcome assessors and analysts blinded) observation study. Seventy-four patients (ASA I–II, aged 18–65 years, BMI < 26 kg/m^2^) undergoing elbow joint release surgery were randomly assigned to either a control group (GA alone) or an observation group (ultrasound-guided brachial plexus block: 20 mL 0.375% ropivacaine + GA). Primary outcome was MMSE scores on pre-op D 1 (preoperative day 1), POD 1 (postoperative day 1), and POD 3 (postoperative day 3). Secondary outcomes included awakening time, extubation time, VAS scores at 1, 6, and 12 hours after extubation and hemodynamic parameters at different time points.

**Results:**

The MMSE scores in the observation group were significantly higher than those in the control group both on the POD 1 (23.06 ± 1.01 vs 20.50 ± 0.51, mean difference 2.56 [95% CI 2.18 to 2.93]; *P* < 0.001) and POD 3 (25.56 ± 0.51 vs 23.36 ± 0.49, mean difference 2.19 [95% CI 1.96 to 2.43]; *P* < 0.001). The postoperative awakening time (3.50 ± 0.56 vs 11.83 ± 1.00 min, mean difference −8.33 [95% CI −8.71 to −7.95]; *P* < 0.001) and extubation time (3.50 ± 0.56 vs 13.08 ± 0.84 min, mean difference −9.58 [95% CI −9.92 to −9.25]; *P* < 0.001) in the observation group were significantly shorter than those in the control group. Furthermore, Visual analogue scale (VAS) scores in the observation group were lower than those in the control group at 1 hour (1.28 ± 0.61 vs 4.92 ± 0.77, mean difference −3.64 [95% CI −3.97 to −3.31]; *P* < 0.001), 6 hours (1.36 ± 0.54 vs 5.67 ± 0.68, mean difference −4.31 [95% CI −4.59 to −4.02]; *P* < 0.001), and 12 hours post-extubation(3.44 ± 0.50 vs 7.67 ± 0.48, mean difference −4.22 [95% CI −4.45 to −3.99]; *P* < 0.001). Hemodynamic stability was superior in the observation group across perioperative phases.

**Conclusions:**

Combining RNB with GA preserves postoperative cognitive function, accelerates recovery, and enhances analgesia in elbow joint release surgery. These findings support RNB as an adjunct to GA to mitigate POCD.

**HIGHLIGHTS**

- Regional nerve block combined with general anesthesia can provide good anesthesia effects.
- Combining regional nerve block with general anesthesia can reduce postoperative pain in patients undergoing elbow joint release surgery.
- Regional nerve block combined with general anesthesia can modify cognitive function in individuals undergoing elbow joint relaxation surgery.

## Introduction

Elbow stiffness, occurring in 5% of trauma patients and occasionally from congenital/acquired causes, often requires surgical release^[1]^. The conventional anesthesia method for elbow joint loosening surgery is general anesthesia, but general anesthesia can lead to shortcomings in postoperative pain management, cognitive function^[2]^. Research had shown that regional nerve block combined with general anesthesia provided good anesthesia effects^[3]^.

The incidence of middle-aged and elderly patients with post-traumatic elbow stiffness is increasing. Their physiological characteristics and anesthesia-related factors may induce postoperative short-term cognitive dysfunction and sleep disorders^[4]^. Surgical pain arises from tissue damage-induced physiological/psychological subjective sensations, potentially exacerbating postoperative recovery^[5]^. Additionally, anesthesia methods and drug selection may impact postoperative cognitive function. Postoperative cognitive dysfunction (POCD) is a serious complication closely related to anesthesia and surgery. In recent years, studies had revealed significant impacts of general anesthesia on cognitive function in elderly patients^[6]^. Regional nerve block was widely used in clinical practice due to its advantages in providing adequate pain relief. Therefore, the study compared the impact of combining regional nerve block with general anesthesia on cognitive function in patients undergoing elbow joint release surgery. No AI was used in the research and manuscript development, the article is compliant with the TITAN Guidelines 2025^[7]^.

## Methods

### Study design and participants

An exploratory study, single-center, single-blind(outcome assessors and analysts blinded) and parallel group RCT was conducted at a tertiary hospital between October 2022 and October 2023. After ethical approval (No. 2024-KY-272(K)) and written informed consent, 74 patients aged 18–65 years (ASA I–II, BMI < 26 kg/m^2^) undergoing unilateral elbow joint release surgery (post-traumatic elbow stiffness) were enrolled. Exclusion criteria included severe comorbidities, allergies to anesthetic agents, or psychiatric disorders. A random assignment (1:1) was used to allocate patients to either the control or observation group using a computer-generated randomization sequence.The control group received general anesthesia (GA) alone,while the observation group received ultrasound-guided brachial plexus block (20 mL of 0.375% ropivacaine, based on standard clinical practice) combined with GA. The allocations were printed and placed in separate sealed envelopes.

### Intraoperative management

Both groups received standardized GA induction (propofol 2 mg/kg, rocuronium 50 mg, sufentanil 30 µg) and maintenance (propofol 4–6 mg/kg/h, remifentanil 0.15–0.3 µg/kg/min). Mechanical ventilation parameters were identical (tidal volume 6–8 mL/kg, FiO2 > 60%). Throughout the anesthesia period, no patients experienced complications such as local anesthetic toxicity. No any adverse events or harms were observed during the trial. If the patient develops adverse reactions such as nerve injury or local anesthetic toxicity during the trial, immediately activate the emergency protocol and terminate the trial. If patients in both groups have a VAS score > 5 at 12 hours after extubation, 2 mg of oliceridine will be administered intravenously as an analgesic rescue measure.

### Observation indicators

Primary outcomes: Cognitive function: Mini-Mental State Examination (MMSE) at preoperative day 1 (pre-op D 1), postoperative day1 (POD 1), and postoperative day 3 (POD 3).

Secondary outcomes: Recovery metrics: Awakening and extubation times. Pain intensity: Visual Analog Scale (VAS) scores at 1, 6, and 12 hours post-extubation. Hemodynamics: Heart rate (HR), systolic/diastolic blood pressure (SBP/DBP) at five intraoperative timepoints (T0: pre-induction; T1: post-induction; T2: during skin incision; T3: the end of surgery; T4: 1 h post-surgery).

### Sample size calculation

The sample size determination was based on the primary outcome of MMSE scores difference between groups, derived from preliminary data (unpublished pilot study, n = 20) showing a mean MMSE scores on postoperative day 3 of 24.45 ± 1.22 in Observation group versus 23.07 ± 2.06 in Control group. Using a Test for the ratio of Two Means with α = 0.05 and β = 0.20 (80% power), the calculated minimum sample size was 33 per group. To account for 10% potential attrition (anesthesia revision/dropout), we recruited 37 participants in each group.

### Statistical analysis

Data were analyzed using SPSS 19.0. Baseline data were compared using chi-square or independent t-tests. Awakening time, extubation time, and VAS scores were compared using independent t-tests. Hemodynamics and MMSE scores over time were analyzed with repeated-measures ANOVA (α = 0.05). If sphericity was violated (Mauchly’s test *P* < 0.05), Greenhouse–Geisser correction and independent t-tests at individual time points were applied.

## Result

### Patient demographics and characteristics

**Figure 1.**
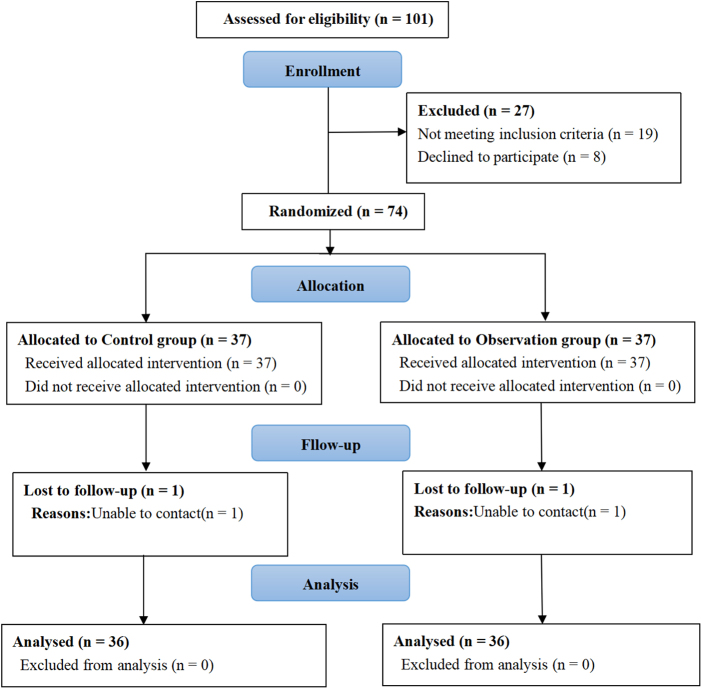
CONSORT diagram of participant selection.

Among 101 initially screened patients, 27 were excluded (19 did not meet inclusion criteria; 8 declined participation). The remaining 74 were randomized equally to control and observation groups (37 each group). Post-randomization, one patient from each group was lost to follow-up, resulting in 36 patients per group completing the study (Fig. 1).

The demographics of the study population and a comparison of control group and observation group are provided in Table 1. All of the variables were similar between the two groups without any significant differences.

**Table 1:** Demographic data and characteristics of patients^a^

Items	Control group (n = 36)	Observation group (n = 36)	t/χ^2^	*P*
Gender/n (%)				
Male	21(58.3)	22(61.1)	0.012	0.850
Female	15(65.2)	14(60.9)		
ASA/n (%)				
I	13(36.1)	16(69.6)	0.023	0.786
II	23(63.9)	20(87.0)		
Age/year	48.11 ± 8.40	50.39 ± 7.11	1.294	0.204
BMI/(kg/m^2^)	22.90 ± 2.29	23.24 ± 1.88	0.808	0.425
Anesthesia time/min	157.50 ± 11.37	156.69 ± 9.22	1.418	0.238
Operative time/min	151.81 ± 10.70	151.67 ± 9.02	1.509	0.223

^a^ Values are number, mean ± SD.

### Primary outcomes

**Figure 2.**
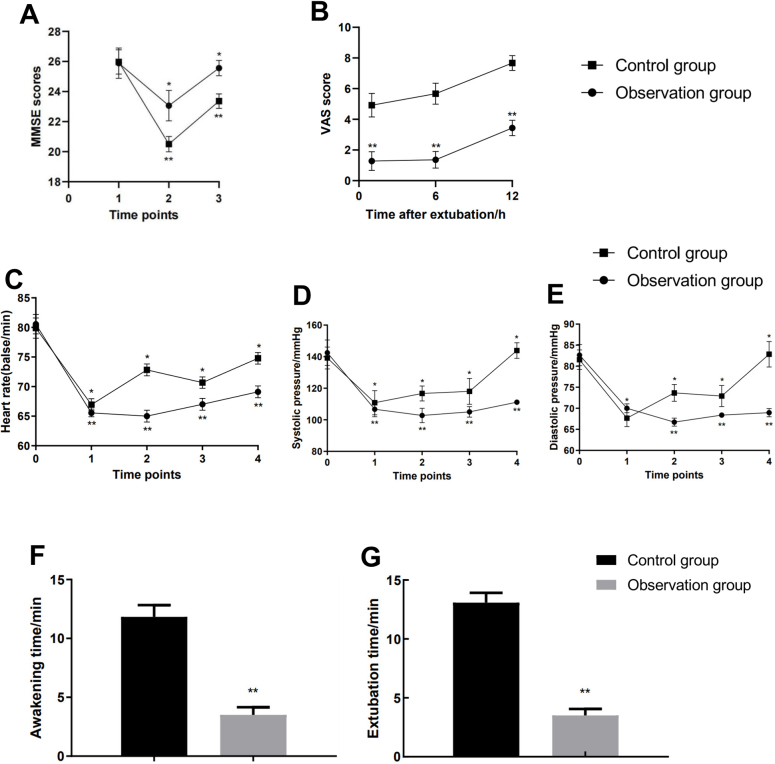
Compares the MMSE scores (A), postoperative VAS scores (B), heart rates (C), SBP (D), DBP (E), awakening time (F) and extubation time (G) at different time points between the two patient groups. Fig. 2A: 1: preoperative day 1 (Pre-op D 1) ; 2: postoperative day 1 (POD1); 3: postoperative day 3 (POD3). Fig. 2C-E: 0 = before anesthesia induction (T0), 1 = after anesthesia induction (T1), 2 = during skin incision (T2), 3 = at the end of surgery (T3), 4 = 1 hour after surgery (T4). **P < 0.001 compared with control group, *P < 0.001 compared with baseline levels. Fig. 2A: 1: preoperative day 1 (Pre-op D1) ; 2: postoperative day 1 (POD1) ; 3: postoperative day 3 (POD3) .Fig. 2C-E: 0 = before anesthesia induction (T0), 1 = after anesthesia induction (T1), 2 = during skin incision (T2), 3 = at the end of surgery (T3), 4 = 1 hour after surgery (T4). **P < 0.001 compared with control group, *P < 0.001 compared with baseline levels.

Figure 2A compares cognitive function between groups using MMSE scores. Pre-op D 1 scores showed no significant difference (observation: 25.89 ± 1.01 vs control: 25.97 ± 0.81, mean difference −0.83 [95% CI −0.51 to −0.35]; *P* = 0.700). POD 1 revealed significantly lower scores in controls (23.06 ± 1.01 vs 20.50 ± 0.51, mean difference 2.56 [95% CI 2.18 to 2.93]; *P* < 0.001). POD 3, controls improved to 23.36 ± 0.49 while observation group reached 25.56 ± 0.51 (mean difference 2.19 [95% CI 1.96 to 2.43]; *P* < 0.001).

### Recovery status and pain relief

Figure 2F–G shows significantly shorter postoperative recovery times in the observation group versus controls, with awakening time (3.50 ± 0.56 vs 11.83 ± 1.00 min, mean difference −8.33 [95% CI −8.71 to −7.95]; *P* < 0.001) and extubation time (3.50 ± 0.56 vs 13.08 ± 0.84 min, mean difference −9.58 [95% CI −9.92 to −9.25]; *P* < 0.001). VAS scores (Fig. 2B) were significantly lower in the observation group at all timepoints: 1 h post-extubation (1.28 ± 0.61 vs 4.92 ± 0.77, mean difference −3.64 [95% CI −3.97 to −3.31]; *P* < 0.001), 6 h (1.36 ± 0.54 vs 5.67 ± 0.68, mean difference −4.31 [95% CI −4.59 to −4.02]; *P* < 0.001), and 12 h (3.44 ± 0.50 vs 7.67 ± 0.48, mean difference −4.22 [95% CI −4.45 to −3.99]; *P* < 0.001).

### Perioperative hemodynamic conditions

Figure 2C–E compares perioperative hemodynamics between groups. At baseline (T0), heart rate (observation: 80.56 ± 1.66 vs control: 79.89 ± 1.72 bpm, mean difference 0.67 [95% CI −0.13 to 1.46]; *P* = 0.810), SBP (142.5 ± 8.06 vs 139.17 ± 6.91 mmHg, mean difference 3.33 [95% CI 0.20 to 6.87]; *P* = 0.064), and DBP (82.64 ± 2.53 vs 81.53 ± 2.34 mmHg, mean difference 1.11 [95% CI −0.03 to 2.26]; *P* = 0.057) showed no intergroup differences (all *P* > 0.05). The observation group demonstrated significantly lower heart rates than controls at T1-T4 (all *P* < 0.001), with maximal reduction at T4 (69.11 ± 1.01 vs 74.78 ± 0.99 bpm, mean difference −5.67 [95% CI −6.14 to −5.20]; *P* < 0.001). SBP was significantly reduced in the observation group at T1-T4 (*P* < 0.001), most notably at T4 (111.17 ± 1.00 vs 143.8 ± 4.94 mmHg, mean difference −32.72 [95% CI −34.40 to −31.05]; *P* < 0.001). DBP became significantly lower in the observation group at T2, peaking at T4 (68.9 ± 0.99 vs 82.83 ± 3.04 mmHg, mean difference −13.86 [95% CI −14.92 to −12.80]; *P* < 0.001).

## Discussion

Elbow joint stiffness following trauma or post-fracture surgery significantly impacts patient recovery^[8]^. This study compared general anesthesia (GA) alone versus regional nerve block combined with GA (RNB + GA) for elbow release surgery. Patients receiving RNB + GA exhibited more stable intraoperative hemodynamics (heart rate, blood pressure), shorter awakening/extubation times, and lower postoperative VAS scores than GA alone. RNB likely enhances localized analgesia, reducing GA and opioid requirements, thereby minimizing central nervous system depression and accelerating recovery^[9]^. Our study found that the patients’ VAS scores decreased by an average of 3.64 points, 4.3 points, and 4.22 points at 1 hour, 6 hours, and 12 hours after extubation, respectively. Reduction > 2 points on the 10-point VAS is widely regarded as clinically meaningful, as it reflects a perceptible improvement in pain relief for patients.

POCD, linked to anesthesia agents, surgical stress, pain, and preoperative factors^[10]^, remains a critical concern. RNB + GA patients demonstrated higher MMSE scores postoperatively, the patient’s MMSE scores increased by an average of 2.56 points and 2.19 points on POD1 and POD3, respectively. An MMSE improvement of 2 points is clinically meaningful, suggesting reduced cognitive impairment. Ropivacaine, a low-toxicity local anesthetic used in RNB, may mitigate neurotoxic effects associated with GA and opioids. POCD pathogenesis involves neuroinflammation and cholinergic signaling disruption. Pain exacerbates inflammatory mediators (e.g., prostaglandins, cytokines), which impair cognition. RNB’s targeted analgesia reduces systemic inflammation and opioid-induced anticholinergic effects, potentially preserving cholinergic function.

Ultrasound-guided RNB prolongs perioperative analgesia, alleviating acute postoperative pain and its neuroinflammatory consequences. This approach may thus indirectly improve cognitive outcomes by minimizing pain-related neurochemical disturbances. Future studies should validate these findings in larger cohorts and explore RNB’s long-term neuroprotective benefits.

### Strengths and limitations

Despite limitations, such as the small sample size limits generalizability, a single-center study with insufficient representativeness, psychological factors influencing sleep, unmeasured confounders, and short-term follow-up, our study offers advantages like combining RNB with GA improves cognitive outcomes compared to GA alone, accelerates recovery, and enhances analgesia in elbow joint release surgery.

## Conclusion

The combination of regional nerve block and general anesthesia is a promising approach for patients undergoing elbow joint release surgery, offering improved pain management, cognitive preservation. Further research is warranted to validate these results in larger, more diverse patient populations.

## Data Availability

Data underlying this study will be shared upon reasonable request to the corresponding author.

## Research registration unique identifying number (UIN)

This research was registered at ClinicalTrials.gov as ID:NO.ChiCTR2400094137.
